# Changing rainfall and humidity within Southeast Texas

**DOI:** 10.1186/s40064-015-1245-7

**Published:** 2015-08-25

**Authors:** Robert Kennedy Smith

**Affiliations:** 8421 Broad Street Apt 607, Mclean, VA 22102 USA

**Keywords:** Southeast Texas, Climate change, Precipitation patterns

## Abstract

Southeast Texas houses a precipitation transition zone between drier conditions to the North and West and some of the wettest parts of the continental U.S. to the East. The Region has seen an increase in its reported normal annual precipitation totals in recent decades. In order to determine if the additional rainfall has been influenced by warming temperatures or is within the variability of the State’s long-term drought cycles, several analyses were performed on historical climate data. The analyses answered several questions: Have global and regional climate change models predicted precipitation increases in Southeast Texas and are future increases expected? Do historical monthly precipitation totals at various sites in the region provide clear trends of wetter conditions that can be discerned from long-term drought cycles? Are rainfall patterns changing with less frequent, heavier rain events? Do the reported increases in annual rainfall actually lead to wetter conditions in the region? Climate models have not predicted larger annual average precipitation totals nor do they forecast increases for Southeast Texas. While recent decades may have seen more rain relative to earlier periods, a combined analysis of observation stations across different parts of the Region shows that long-term trends are dependent on when the data is selected relative to a drought cycle. While some stations show larger amounts of rain falling during fewer days, these trends do not hold across all periods. An examination of hourly data does not show an increase in extreme rainfall events or a decrease in the number of hours during which rain has fallen. Even though rainfall has not decreased, average relative humidity has fallen. This suggests that the area is drying even with steady or increasing amounts of rain.

## Background

Each decade, the National Oceanic and Atmospheric Administration publishes its revised list of 30-year climate normals for several thousand locations across the United States (NCDC 2012). Sites within Southeast Texas, the area housing the Houston metropolitan area, have shown increases in normal annual precipitation (Table 1). Additional studies have also shown that the Region, on average, has experienced a 10–15 % increase in annual rainfall when comparing 1991–2012 averages to 1901–1960 baseline estimates (Melillo et al. 2014). In order to better discern if the reported increases may be significant and attributable to anthropogenic activity or fall within the realm of the Region’s long-term drought cycle, a survey of climate literature relevant to Southeast Texas was conducted to provide contextual relevance for the statistical analysis performed in this study.

**Table 1:** NOAA 30-year annual precipitation normals for two locations in Southeast Texas

Time period	City of Houston normal rainfall (in.)	Barker (NW Houston) normal rainfall (in.)
1951–1980	44.77	40.52
1961–1990	46.07	42.39
1971–2000	47.84	46.96
1981–2010	49.77	48.66

**Fig. 1:**
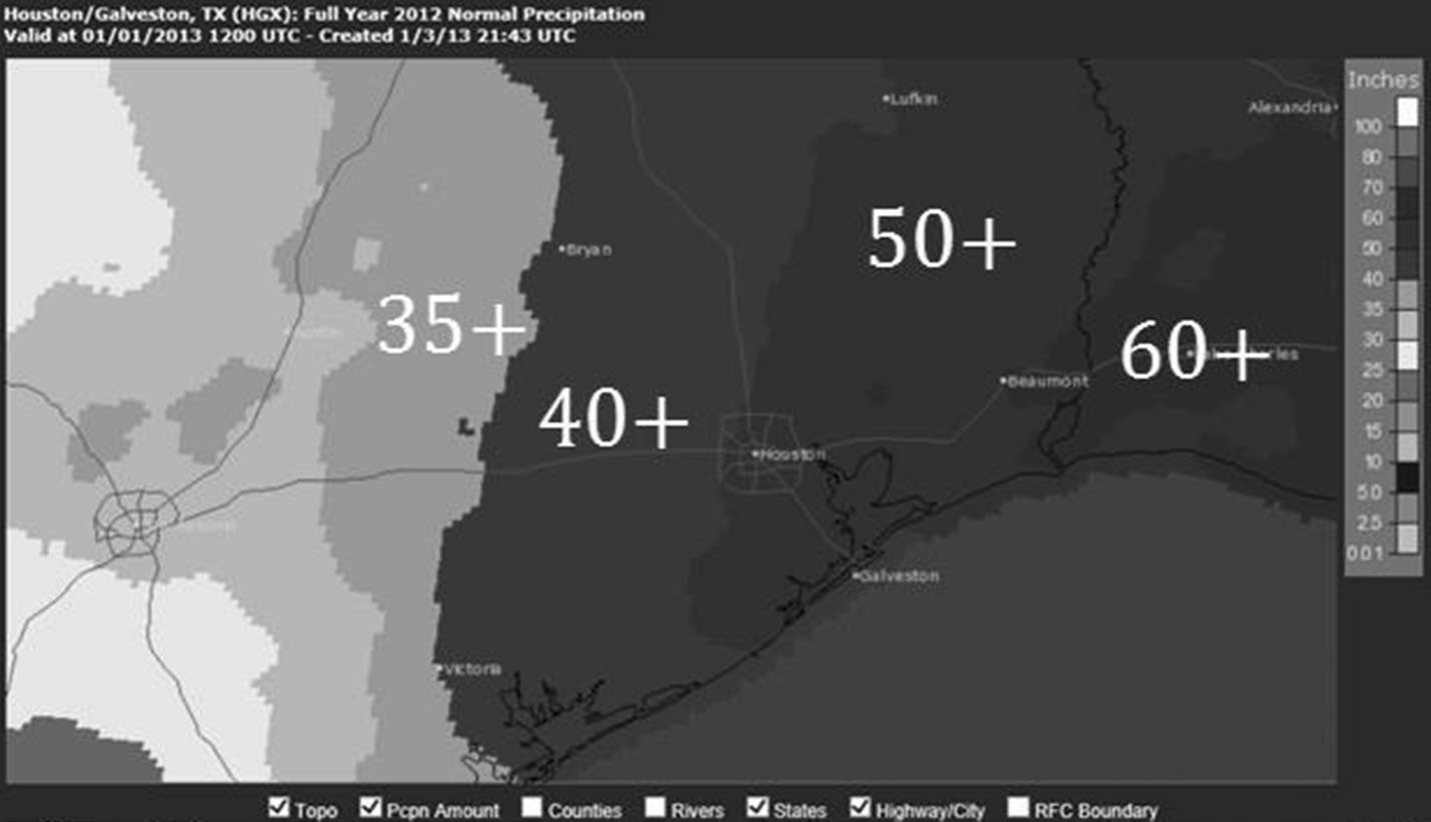
Normal annual precipitation over Southeast Texas, 1981–2010 (in.) Source: National Weather Service, Advanced Hydrologic Prediction Center

In Southeast Texas, precipitation patterns are heavily influenced by mesoscale events associated with the Gulf of Mexico (Ohashi and Kida 2002), and average rainfall increases dramatically moving from the Northwest towards the Southeast. According to the National Weather Service’s Advanced Hydrologic Prediction Service, the metropolitan area of Houston receives approximately 50 in. of rain annually, with 5-inch decreases in average totals occurring over east-to-west partitions that are 20 miles wide (NOAA, AHPS 2014) (Fig. 1). Unlike other southeastern cities, year-to-year rainfall totals can vary heavily. While Houston and Atlanta share similar average precipitation profiles, with each city receiving its 50-inch total at relatively uniform rates throughout the year, larger variations in annual totals are more prevalent in Houston. In 2007, as Atlanta was reeling from a severe drought, that city received nearly 32 in. of precipitation during the calendar year (Johnston 2007); in the past 30 years, Intercontinental Airport’s (IAH) rain gauge has been below this level three times (NCDC 2012). The coefficients of variation for Houston and Atlanta, the standard deviation of annual rainfall totals from 1970 to 2012 divided by the mean, is 0.26 and 0.17, respectively. Area soil moisture content between 2003 and 2012 has been reduced by an average rate of 2.3 cm annually due to recent dry conditions (Famiglietti and Rodell 2013). Ten years earlier, Houston was inundated by Tropical Storm Allison (Berger 2011), causing 2001 precipitation totals to exceed 70 in. at IAH and 80 in. at Hobby Airport.

Comparisons of the models projecting precipitation trends under global climate change scenarios do not yield high-confidence projections for the Houston metropolitan area, as long-term rainfall forecasts house more uncertainty than temperature projections (Wentz et al. 2007; Christensen et al. 2013). The fifth assessment from the Intergovernmental Panel on Climate Change (IPCC) generally projects precipitation increases several hundred miles to the northeast of Houston and decreases several hundred miles to its southwest over the next 80 years (Christensen et al. 2013). While the models used in the IPCC analysis capture large-scale phenomena affecting the Region, such as projected changes in the El Niño-Southern Oscillation, the North Atlanta Oscillation, the Pacific North American pattern, and the Pacific Decadal Oscillation, they do not concentrate on potential impacts to mesoscale events such as the summertime afternoon sea-breeze that provides Southeast Houston with frequent scattered thunderstorms (Shepard et al. 2010). High uncertainty associated with large-scale weather pattern forecasts and the lack of detailed mesoscale impact modeling create low-confidence rainfall projections for Southeast Texas. In NOAA’s regional climate change assessment, the means of several global and regional model results are shown for southwestern Louisiana and extreme southeastern Texas (NOAA, NESDIS 2013). The mean global model results show a 6–9 % decrease in rainfall over the Region by the end of the century. Meanwhile, the averaged results of the regional models show increases in precipitation in each of the four seasons, with annual rates forecasted to be 6–9 % higher than current levels although the projections are shown to be low-confidence estimates. Another study showed ambiguous effects of a warmer climate on Southeast Texas, using model averages to forecast a small gain in annual rainfall (Jiang and Yang 2012).

As the population of Houston has grown dramatically since the 1950s (not coincidentally during the time period in which air conditioning usage became widespread), the city’s urban heat island effect has grown in area due to new development, spreading to the North and West. A comprehensive study of precipitation patterns downwind of the urban heat island, within the urban area, and in areas influenced by the urban core (Houston’s western and northern suburbs), shows a dramatic increase in afternoon and early-evening rain events during the summer months (Burian and Shepherd 2005). Total summertime rainfall accumulation in the early 1990s often exceeded 1950s levels by more than 25 %. Precipitation increases during the winter months were less dramatic but still present. Burian and Shepherd’s overall trends may be affected by the reference time period chosen; the 1950s were an extremely dry period for the State of Texas (Morello 2011), but the findings still show how small-scale impacts can have profound climatic effects that are not reflected in global or most regional projections.

This study examines whether the reported increases in Southeast Texas’ annual rainfall show trends independent of the long-term drought cycle. Historical monthly rainfall data for four stations in different areas in the Houston metropolitan area is analyzed for significant trends. Rainfall data at two of the stations with complete records extending beyond the Texas drought in the 1950s is also reviewed, with trends during this expanded timeframe compared to the 1970–2012 period. In order to determine if the characteristics of Houston’s rainfall have changed over time, daily and hourly rainfall records are analyzed. At the Region’s only official observation station where hourly precipitation data was available from 1970–2012, records were used to determine if time-related changes could be found in the frequency of heavy and extreme precipitation events, the average number of hours annually during which rain fell, and the average rate of rainfall when it did occur. For the other stations without hourly information, one-day rainfall trends were reviewed for the 1970–2012 period and the extended period that included the 1950s drought. Finally, monthly relative humidity trends at the official observation station were analyzed and compared with rainfall trends for that location.

## Method

**Fig. 2:**
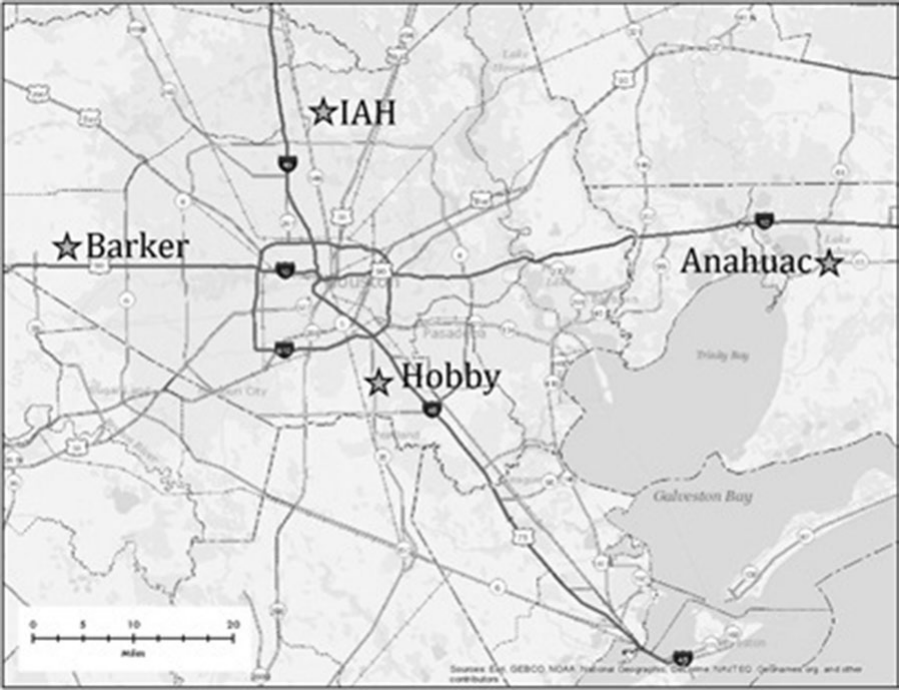
Observation point locations in Southeast Texas

Historical monthly rainfall totals and daily precipitation data were collected from four stations with comprehensive records that roughly represent the geographic edges of the Houston metropolitan area: Intercontinental Airport (IAH), William Hobby Airport (Hobby), Houston Barker (Barker), and Anahuac (Fig. 2). The Global Historical Climatology Network (GHCND) identification numbers are USW00012960, USW00012918, USC00414313, and USC00410235, respectively. IAH represents the northernmost point and is 24 miles NNW of Hobby, 25 miles NE of Barker, and 46 miles WNW of Anahuac. Barker is the westernmost and driest observation station, located 29 miles WNW of Hobby and 66 miles west of Anahuac. Anahuac, the easternmost and wettest station lies 30 miles ENE of Hobby. All data originated from the National Climatic Data Center (NCDC) and was obtained by examining daily records to ensure that missing days with zero values were not averaged into monthly summaries, creating inaccuracies. The data consisted of 516 monthly rainfall totals from 1970 through 2012, since Intercontinental Airport opened in 1969. Days in which measurements did not appear were populated with data from the nearest observation stations. Since each observation station had few unrecorded days over the 43-year period, the effect of substitution was negligible. Average annual rainfall (1970–2012) for Barker, IAH, Hobby, and Anahuac was 47.18, 48.85, 54.05, and 55.01 in., respectively. The Hobby and Anahuac stations accumulate more rainfall during the summer months as subtropical convection prevails. Hobby and Anahuac recorded, on average, about 4 more in. of precipitation during June, July, and August, than did IAH and Barker. The monthly observations were entered into the statistical analysis software program R as time series data in order to adjust for seasonality. This was done by subtracting the monthly seasonal component leaving observations containing the trend and random components. Even though Southeast Texas has relatively uniform long-term monthly normal rainfall patterns (NCDC 2012), late spring and early fall tend to be wetter than winter and early spring—in eastern parts of the Region wetter conditions persist throughout the summertime as noted above. By subtracting the monthly expected variation—positive values for historically wet months and negative values for dry months—the cyclical variation is eliminated and analysis can be conducted on long-term trends. The adjusted data was then analyzed for statistically significant changes over time.

Trends in the number of rainy days for the same four locations are also examined. In order to determine whether the statistically significant findings at these stations can be maintained even within the long-term drought cycles that make Houston’s climate highly variable, two stations with longer data records are examined. Trends from the extended timeframe, which includes a multiyear drought, are compared to the 1970–2012 base period.

Intercontinental Airport is the official observation site for Southeast Texas and houses the Region’s only continuous record of hourly rainfall. The 1970–2012 base period was used to determine if less frequent, heavier downpours have increased. Using hourly data can provide added precision in identifying trends relative to the daily totals and is of potential use for policymakers concerned with flooding associated with extreme, brief events. After the completion of these analyses, a linear regression of seasonally-adjusted monthly relative humidity values at IAH during 4 day parts is presented and compared with observed precipitation trends.

## Results and discussion

Rainfall pattern trends at the four observations stations are determined using linear regression analysis, regressing adjusted monthly rainfall totals over time. In order to ensure that the least-squares estimators are unbiased and efficient, the Breusch–Pagan test was employed. This test determines if heteroscedasticity is present in a linear regression model and showed that three out of the four stations did not exhibit variance patterns in which heteroscedasticity could be verified, however it did show increasing month-to-month variability at the Barker station. Therefore, a weighted least-squares regression was used to measure Barker rainfall trends, the weights expressed as $\frac{1}{{(si)^{2} }}$ with the denominator of the expression representing the square of the expected monthly standard deviation. The finding that monthly rainfall variability at the Barker station is increasing over time is notable, showing that dry months in the area are becoming drier at the same time that wet months are becoming wetter. This increased volatility agrees with several assessments that climate change may result in strongly entrenched wet and dry patterns, creating more frequent weather extremes. Nonetheless, the weighted least-squares regressions did not result in a statistically significant overall trend at any of the four observation stations (Table 2). Monthly rainfall total trends over the past 43 years in Southeast Texas are not discernable even though adjustments to climate normal values have shown the area became wetter.

**Table 2:** 1970–2012 rainfall trends in Southeast Texas

Measurement	Rainfall in inches (weighed) least-squares equation (*y* = inches of rain, *x* = number of months since Dec 1969)	Decadal trend (in./decade)	P-value (slope ≠ 0)
IAH seasonally-adjusted monthly rainfall (in.)	y = 0.000x + 4.07	0.00	–
Anahuac seasonally-adjusted monthly rainfall (in.)	y = 0.000x + 4.61	0.01	0.93
Hobby seasonally-adjusted monthly rainfall (in.)	y = −0.001x + 4.75	−0.11	0.33
Barker seasonally-adjusted monthly rainfall (in.)	y = 0.001x + 3.80	0.06	0.55

The number of days per year in which varying levels of precipitation fell was also regressed against time at each of the four observation stations chosen above. Days during which at least 1.95 in. of rain fell (50 mm), 0.4 in. (10 mm) or more of rain fell, or any measurable precipitation occurred were recorded. Three out of the four stations, IAH, Hobby, and Anahuac, showed no statistically significant trends in annual daily rainfall events over time. However, since 1970, Barker has shown a dramatic decrease in the average number of days during which measurable precipitation fell (there were no significant trends at that station in the number of days experiencing relatively large rain events). The least-squares regression of the number of days receiving rain is given by the following equation:

1 $$y_{(any)} = - 0.51x + 87.2$$

where *y*_(*any*)_ is equal to the expected number of days per year during which measurable rain will fall and *x* is the number of years since 1969. For the 43-year period, the trend equates to a loss of 5 days of measurable rain each decade; 22 fewer rainy days each year can be expected in 2012 than in 1970 even though expected total monthly and annual precipitation has not decreased.

In order to determine how a chosen time period affects long-term trends, the data sets were expanded by 22 years, and observations were now included from 1948 to 1969, increasing the total number of monthly observations to 780. Station records before 1948 were incomplete. The two stations with the most comprehensive records were chosen: Hobby and Barker. As noted, Hobby represents the wetter area southeast of downtown, while Barker is located west-northwest of downtown and is among the driest areas of the city. Since three of the ten driest years on record for the State of Texas occurred during the 1950s, the revised observation period incorporates a long-term drought event early in the period of record. Extreme Southeast Texas did not experience conditions nearly as dry as the rest of the state during the 1950s, leading to more severe deficits at the Barker station relative to Hobby (NOAA, NWS 2013b). In the 10-year period between 1948 and 1957, Barker experienced 5 years with rainfall below 26 in. Hobby had no years in which rainfall was below 28 in., and only 2 years that recorded <30 in. of rain.

As in the shorter period, the time series data for both observation stations was seasonally adjusted and a weighted least squares regression was required to address heteroscedasticity in the monthly observations at the Barker station. Table 3 shows rainfall trend results. In contrast to the trends estimated during the 1970–2012 period, all seasonally-adjusted monthly precipitation trends are statistically significant at the 10 % level.

**Table 3:** 1948–2012 rainfall trends and comparisons at Hobby and Barker Stations

Measurement	Rainfall in inches (weighed) least-squares equation (*y* = inches of rain, *x* = number of months since Dec 1947)	Decadal trend (in./decade)	P-value (slope ≠ 0)
Hobby seasonally-adjusted monthly rainfall trends (in.)	y = 0.001x + 3.91	0.11	0.08
Barker seasonally-adjusted monthly rainfall trends (in.)	y = 0.002x + 3.00	0.20	0.00
Surplus seasonally-adjusted monthly rainfall at Hobby relative to Barker (in.)	y = −0.006x + 0.84	−0.07	0.08

While the slopes measuring the trends appear small, the period of record, 780 months, allows for a pronounced impact. For example, during the first year on record, the Barker station could expect approximately 36 in. of precipitation (the sum of 12 seasonally adjusted months), much less than the 1981–2010 average. If the trend is applied to end of the observation period, the average year receives over 50 in. of rain, which is close to the average annual rainfall of 48 in. it has received since 2000. At Hobby, the trend is more gradual. This is not surprising since its drought years during the 1950s were not as severe as what was experienced at Barker. Accordingly, the seasonally-adjusted surplus monthly rainfall at Hobby was cut dramatically over the 65-year period from nearly 1 in. to around one-third of an inch, on average, each month.

The number and type of daily precipitation events over the extended observation period were also explored. For Hobby, the only significant trend was an increasing number of days during which more than 0.4 in. (10 mm) of rain was recorded: the station gained slightly <1 day per decade when this amount could be expected. The equation is given by the following:

2 $$y_{(0.4)} = 0.09x + 32.68$$

where *y*_(0.4)_ is equal to the expected number of days per year during which 0.4 in. or more rain will fall and *x* is the number of years since 1947. During the shorter period of record, the number of days during which large amounts of rain fell did not show a definitive trend at either station. Since the extended period started with severe drought at Barker, precipitation event trends are now quite different relative to the 1970–2012 period. Least-squares trends for the number of days in which large amounts of rain fell were statistically significant at the 5 % level when using the 1.95-inch and 0.4-inch thresholds, and are shown in the following two equations:

3 $$y_{(1.95)} = 0.05x + 2.79$$

4 $$y_{(0.4)} = 0.12x + 28.45$$

where *y*_(1.95)_ and *y*_(0.4)_ are equal to the expected number of days per year during which 1.95 and 0.4 in. or more of rain will fall, respectively, and *x* is the number of years since 1947. Despite the dramatic trend in the 1970–2012 data showing the Barker station losing 5 days of measurable precipitation per decade, the longer period did not show a statistically significant trend indicating any decrease or increase in the total number of days receiving measurable precipitation.

Analysis of hourly rainfall data at IAH did not yield strong patterns associated with time. From 1970 to 2012, the average number of hours during which 0.25 in. or more of rain fell at Intercontinental airport was 50. In 1988, that amount of hourly rain was recorded only 24 times, while in 1997 at least 0.25 in. of rainfall occurred during 76 hourly observations. The *p* value of the least-squares estimate of the number of occurrences of that amount of rain regressed against time was large and thus no trend is conclusive. With respect to the number of hours during which any measurable precipitation was reported, IAH averaged 423 with a recorded median of 407 h. The calculated trend, showing a decrease of nearly 18 h of measurable precipitation per decade, missed the 10 % statistical significance threshold. Rainfall totals occurring in hours reporting 0.02 in. or more of rain was divided by the number of hours reporting the occurrence, producing an average of hourly rainfall rates that excluded records of 0.01 in. of rain or drizzle. The average of 0.15 in. of rain per hour did show a statistically significant positive trend at the 10 % level, but the trend is small; it increased by 0.04 in. per century. Of potential interest to policymakers involved in flood planning are trends involving the number of hours during which extreme rainfall occurred. All years since 1970 have seen at least 1 h when 1 in. or more of rain fell and all but 1 year contained fewer than ten events. The average for the 1970–2012 time period is 5.4 h per year. While there was a positive trend in the frequency of these events, low annual totals give the outlying wet year 2001 oversize influence. Tropical Storm Allison crossed the Houston metropolitan area twice in early June with devastating floods (Berger 2011). If the 14 hourly one-inch or greater rainfall events in 2001, the majority of which occurred in June due to Allison, are excluded from the 43-year period, there is no increase or decrease in extreme hourly rain events. Overall, there is little to suggest that hourly rainfall is becoming heavier and less frequent although trends may become statistically significant if future years entrench existing patterns.

**Fig. 3:**
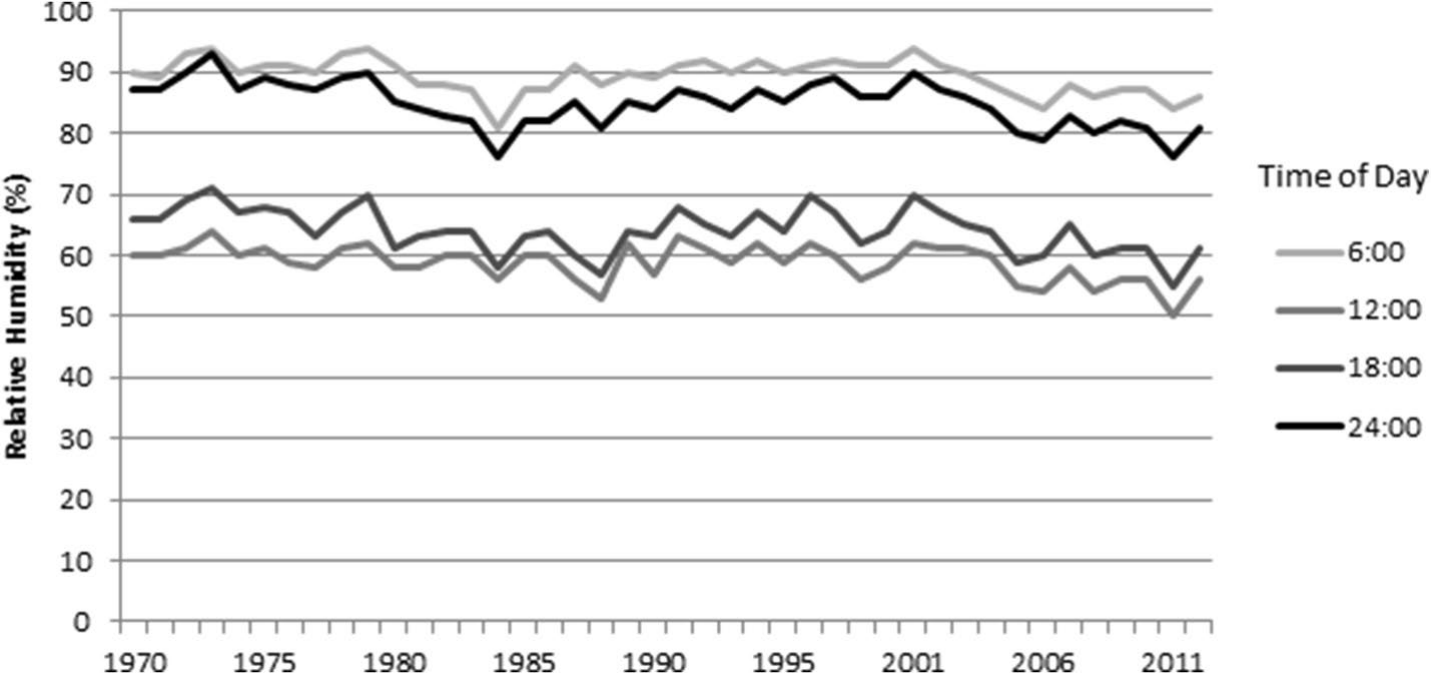
Average annual relative humidity measurements at 06:00, 12:00, 18:00 and 24:00

At IAH, four hourly relative humidity (RH) measurements from each day were averaged into monthly summaries by the National Climatic Data Center (NOAA, NWS 2014). Daily RH values are calculated automatically at the airport based on readings of other parameters (such as dew point and air temperature) and have been recorded at the same location continuously since 1969. The readings occurred at 06:00, 12:00, 18:00, and 24:00 Central Standard Time. The data consisted of 516 monthly records that were seasonally adjusted using the method described earlier. Seasonal RH values were also examined: winter readings were obtained from the average of December, January, and February data, and summer readings were from averaged June, July, and August observations. No heteroscedasticity was observed. Annual average RH is presented graphically in Fig. 3. Using least-squares regression analysis, all of the seasonally-adjusted relative humidity measurements show a statistically significant decrease (Table 4). Although the decreases were not dissimilar in magnitude, the midnight hour measurements showed the largest decrease of 1.6 % RH per decade. Following this trend, on average, the midnight hour in the early 1970s at IAH would expect RH readings near 90 %, while current-day readings would average just above 80 %. Summertime drops in RH were larger than wintertime decreases. The 06:00 RH reading during the warmest months in Houston showed a decreasing decadal trend of 1.8 % points, which is the largest drop calculated. During the winter, RH decreases for each period hovered near 1 % per decade.

**Table 4:** 1970–2012 monthly relative humidity trends at intercontinental airport

Measurement	Rainfall in inches least-squares equation (*y* = RH*, x* = number of months since Dec 1969)	Decadal trend (%/decade)	P-value (slope ≠ 0)
IAH seasonally-adjusted relative humidity at 0:00 (%)	y = −0.013x + 87.97	−1.56	0.00
IAH seasonally-adjusted relative humidity at 6:00 (%)	y = −0.012x + 87.95	−1.44	0.00
IAH seasonally-adjusted relative humidity at 12:00 (%)	y = −0.009x + 61.03	−1.08	0.00
IAH seasonally-adjusted relative humidity at 18:00 (%)	y = −0.010x + 66.66	−1.20	0.00

## Conclusions

Houston has high yearly rainfall variability relative to other wet areas in the eastern U.S. In September 2013, the National Weather Service office in Houston published a study showing that Southeast Texas (climate division 8) ranked first out of the 344 climate regions for largest rainfall deficit as measured in inches of rain from October 2008 through August 2013 (NOAA, NWS 2013a). For that period, IAH is 53.33 in. below its 1981–2010 normal accumulation and Hobby is 59.37 below its average. Thus the dataset for this study ends with a very dry period for Southeast Texas. Due to the recent dryness, the seasonally-adjusted data from all four observation stations over the 1970–2012 period did not show trends indicating the area is becoming wetter. The number of rainy days at each observation location fluctuated depending on the time period considered, although a dramatic recent trend was observed at Barker observation station which has lost one rainy day, on average, every 2 years over the past 43 years. When the analysis is extended to include the 1950s drought, both Hobby and Barker showed increases in monthly total rainfall, with Barker gaining about two-tenths of an inch of rain per month each decade. Despite the recent decrease in rainy days at Barker, observations over the longer time period did not show a similar trend. The analysis shows that monthly and annual precipitation trends during recent decades have been dominated by long-term drought cycles and that changes in the Region’s annual rainfall normal values (Table 1) are more reflective of the time period chosen than a wetter climate.

Hourly observations at the area’s official reporting station fail to demonstrate with reasonable confidence that less frequent, more intense annual rainfall events are occurring now than in the past. In contrast to the numerous statistically insignificant findings associated with rainfall, monthly relative humidity levels have shown a clear decrease. Since the period of record for these readings is 43 years, it is possible that changing long-term weather patterns will reverse the declines, although they have not been associated with less overall precipitation at the reporting station and therefore cannot be associated with drought. This suggests that Houston will dry in the future without larger increases in average annual rainfall amounts.

## References

[CR1] Berger E (2011) Progress and lessons 10 years after Tropical Storm Allison. The Houston Chronicle. **(Print)**

[CR2] Burian SJ, Shepherd JM (2005). Effect of urbanization on the diurnal rainfall pattern in Houston. Hydrol Process.

[CR3] Christensen JH, Kanikicharla KK, Aldrian E, An S, Cavalcanti I, Castro M, Dong W, Goswami P, Hall A, Kanyanga J, Kitoh A, Kossin J, Lau N, Renwick J, Stephson D, Xie S, Zhou T (2013) Climate Phenomena and their Relevance for the Future Regional Climate Change. Climate Change 2013: The Physical Science Basis. Contribution of Working Group I to the Fifth Assessment Report of the Intergovernmental Panel on Climate Change. Cambridge University Press, London

[CR4] Famiglietti JS, Rodell M (2013). Water in the balance. Science.

[CR5] Jiang X, Yang ZL (2012). Projected changes of temperature and precipitation in Texas from downscaled global climate models. Clim Res.

[CR6] Johnston L (2007) Rainfall Q&A. The Atlanta Journal Constitution. **(Print)**

[CR7] Melillo JM, Richmond TC, Yohe GW (2014) Climate change impacts in the United States: the third national climate assessment. U.S. Global Change Research Program

[CR8] Morello L (2011) Some climatologists worry that Texas’ mega-drought could endure for years. The New York Times. **(Print)**

[CR9] National Climatic Data Center (NCDC) (2012) Climate data online. http://www.ncdc.noaa.gov/cdo-web. Accessed 25 Nov 2013

[CR10] NOAA, National Environmental Satellite, Data, and Information Service (2013) Regional climate trends and scenarios for the U.S. National Climate Assessment. Part 2: climate of the Southeast U.S. NESDIS 142-2

[CR11] NOAA, National Weather Service, Advanced Hydrologic Prediction Service (2014) Houston/Galveston full year normal precipitation. http://www.water.weather.gov/precip. Accessed Jan 2014

[CR12] NOAA, National Weather Service, Houston/Galveston, TX Weather Forecast Office (2013a) Drought conditions compound since 2008. http://www.srh.noaa.gov/hgx. Accessed Dec 2013

[CR13] NOAA, National Weather Service, Houston/Galveston, TX Weather Forecast Office (2013b) Houston’s annual top 10 list. http://www.srh.noaa.gov/hgx. Accessed Oct 2013

[CR14] NOAA, National Weather Service, Houston/Galveston, TX Weather Forecast Office (2014) Houston extremes, normals, and annual summaries. http://www.srh.noaa.gov/hgx/?n=climate_iah_normals_summary. Accessed Dec 2014

[CR15] Ohashi Y, Kida H (2002). Local circulations developed in the vicinity of both coastal and inland urban areas: numerical study with a mesoscale atmospheric model. J Appl Meteorol.

[CR16] Shepard JM, Carter M, Manyin M, Messen D, Burian S (2010). The impact of urbanization on current and future coastal precipitation: a case study for Houston. Environ Plan.

[CR17] Wentz FJ, Ricciardulli L, Hilburn K, Mears C (2007). How much more rain will global warming bring?. Science.

